# Hormonal Contraception and Bone Health in Adolescents

**DOI:** 10.3389/fendo.2020.00603

**Published:** 2020-08-21

**Authors:** Laura K. Bachrach

**Affiliations:** Stanford University School of Medicine, Stanford, CA, United States

**Keywords:** oral contraceptives, depo medroxyprogesterone (DMPA), adolescents, fractures, bone accrual, bone mineral density

## Abstract

Hormonal contraception is prescribed commonly to adolescents for myriad indications from pregnancy prevention to treatment for acne, hirsutism or dysmenorrhea. Although use of these hormones generally has no effect or benefits bone health in mature premenopausal women, the same may not be true for adolescents. The teen years are a critical period for acquiring peak bone strength. Sex hormones, growth hormone, and insulin-like growth factors (IGFs) interact to modulate the changes in bone size, geometry, mineral content, and microarchitecture that determine skeletal strength. Combined oral contraceptives (COCs) and intramuscular depo medroxyprogesterone (DMPA) can compromise the expected gains in adolescence by altering estrogen and IGF concentrations. Use of these medications has been associated with slower accrual of bone mineral density (BMD) and increased fracture risk in some studies. Far less is known about the skeletal effects of the newer long acting reversible contraceptives (LARCs). This review takes a critical look at the gaps in current knowledge of the skeletal effects of COCs, DMPA, and LARCs and underscores the need for additional research.

## Introduction

Adolescence is a critical period for establishing bone health when nearly half of peak bone mass is acquired. The most rapid bone mineral accrual occurs about 6 months after peak height velocity during the adolescent growth spurt and continues even after final height is reached (1). Throughout the teen years, the skeleton is changing in bone mass, geometry and microarchitecture, the key determinants of bone strength (2–4). The age at which peak bone mass is achieved varies for the hip, spine and other sites, but 90–95% occurs by age 18 in females (1).

Sex steroids and growth hormones modulate bone size, mineral acquisition and geometry throughout puberty. Alterations in these hormones during this critical period of skeletal development can have lasting effects on peak bone strength acquired by early adulthood. Some hormonal contraceptives alter estrogen, growth hormone (GH) and insulin-like growth factor 1 (IGF-1) concentrations depending upon the dose and route of administration of synthetic estrogens or progestins (5). The skeletal effects of these medications also vary depending upon the sexual maturity and lifestyle habits of the patient. Finally, hormonal contraceptives can have differing effects on females with normal reproductive function and those with hypogonadism.

This chapter reviews current knowledge of the skeletal effects of hormonal contraception with an emphasis on use during adolescence. Studies of mature women provide reassurance that these medications have no effect or may benefit bone health. Bone mineral density (BMD) is similar or greater and fracture rates are similar or reduced in women who have used combination oral contraceptives (COC) as compared to non-users (6). By contrast, studies of adolescents have reported compromised rates of bone mineral accrual with hormonal contraceptive use, especially in the first 3 years post menarche (6). Addressing uncertainties about the extent and reversibility of these skeletal effects is important given the widespread use of hormonal contraceptives not only to prevent pregnancy, but to reduce acne or hirsutism, to regulate heavy or painful menses or to treat amenorrhea (7–10). This chapter focuses primarily on the skeletal effects of hormonal contraceptives in adolescents with normal reproductive function. Use of these formulations as hormone replacement for hypothalamic amenorrhea associated with anorexia nervosa or the female athlete triad will be discussed only briefly.

## Endocrine Control of Adolescent Bone Development

Bone growth, modeling and remodeling are modulated by estrogen, androgen, growth hormone (GH) and IGF-1 (5). GH secreted by the pituitary stimulates IGF-1 production in myriad cells with most of the circulating IGF-1 produced in the liver. IGF-1 increases bone formation by stimulating osteoblast differentiation. Estradiol inhibits bone resorption by increasing osteoclast apoptosis and reducing apoptosis of osteoblasts. Progesterone acts in partnership with estrogen, having an anti-resorptive effects on bone (11). *In vitro*, progesterone has a stimulatory effect of osteoblast differentiation at physiologic concentrations and an inhibitory effect at pharmacologic amounts (11).

Production of GH, IGF-1, and sex steroids increases during puberty with varying concentrations during the menstrual cycle. The rise in estradiol levels between the early follicular phase and mid-cycle stimulates an increase in GH and IGF-1 (12). Conversely, when exogenous estradiol is administered orally, the IGF-1 response to exogenous GH is blunted (13). This effect of exogenous estrogens on IGF-1 may have an impact on bone metabolism, especially during adolescence as discussed below.

Bone strength is determined by bone mass, geometry and microarchitecture, all of which evolve throughout adolescence. Estrogens regulate pubertal changes in bone geometry, stimulating periosteal bone apposition while inhibiting endocortical bone resorption (2). The effect of estrogen on bone is biphasic. In early puberty, low concentrations of estrogen stimulate periosteal apposition while higher concentrations in later puberty inhibit this process. The net result of these processes is an increase in the diameter of bone and in diaphyseal BMD.

High resolution peripheral quantitative computed tomography (HRpQCT) has provided additional insights into changes in bone microarchitecture in adolescence. Gains in trabecular and cortical volumetric BMD, cortical area, cortical porosity, and cortical and trabecular thickness differ in males and females and are modified by physical activity (3). Bone morphology evolves during puberty with alternations in the density and morphology of trabeculae in the radius and tibia in a direction favoring bone strength (4). Lean body mass proved to be the strongest correlate of changes in trabecular morphology, underscoring the importance of muscle-bone interactions. The osteocyte within bone is an important “mechano-sensor,” signaling the bone-building osteoblast in response to skeletal loading. Estrogen increases the sensitivity of the osteocytes to loading stimuli, thus influencing gains in bone strength with activity in puberty (14).

## Combined Oral Contraceptives (COCs) and Bone Mineral Density in Healthy Adolescents

In mature, premenopausal women, COCs have been shown to have no effect or to benefit skeletal health as assessed both BMD or fracture rates (6). A review of 13 studies in women over age 30 using varying low-dose COCs reported a positive effect in 9 studies and no effect in 4 studies (15). Other studies have examined whether the skeletal effects vary by dose of ethinyl estradiol (15 vs. 20 mcg) (16) or by differing progestins (drospirenone or gestodene) (17). In both studies, change in spine BMD did not differ between COC users and controls not using hormonal contraception. A third study found no difference in percent changes in spine, hip or total body BMD over 3 years between current COC users and controls (ages 18–39 years) not using hormonal contraceptives (18).

In contrast to observations from adult women, studies in teens indicate that COC use in adolescence can compromise bone mineral acquisition, especially in the first 3 years post menarche. Initial reports noted lower rates of bone mineral accrual in teens using low-dose (20 mcg ethinyl estradiol) COC formulations when compared with controls not taking hormonal contraceptives. A 1 year study found smaller mean gains in BMD in 79 teens (aged 12–18 years) taking a low-dose COC than in 107 non-user controls (19). Spine BMD increased by 2.3% (95% CI 1.49, 3.18) in users as compared with 3.8% (95% CI 3.11, 4.57) in controls (*P* < 0.001). Gains in femoral neck BMD were also significantly lower (0.3% vs. 2.3%, respectively, *p* = 0.03). A second study found significantly lower bone mineral acquisition in 67 adolescents (ages 12–19 years) using a low-dose COC as compared with non-users (20). These findings are consistent with those reported for young adult women (aged 19–22) taking low-dose COCs whose spine BMD was unchanged over 5 years while non-users gained 7.8% (21).

Other studies have reported skeletal effects of COCs in adolescents at doses ranging from 15 to 35 mcg of ethinyl estradiol (22). A 2 year observational study of adolescents (aged 16–19 years) comparing never to ever COC users found that controls had significantly greater gains in average total hip BMD than did those who had ever used COCs (difference = 0.012 gm/cm^2^; 95% CI +0.001, +0.023 g/cm^2^/2 y). Never-users also had greater gains in femoral neck and spine BMD that were not significantly greater than in COC-users. In the larger cohort (ages 16–24), adjusted BMD changes were not significantly different in those taking COCs containing less than or >/= 30 mcg ethinyl estradiol.

Available COC formulations vary not only in estrogen content but in the number of days of active and placebo tablets designed to induce a withdrawal period either monthly or every 3 months. The skeletal effects of these differing formulations were evaluated in a 12 month, multicenter, open-label, controlled study of 829 adolescent girls (ages 12–18) (23). Teens requesting COCs were randomized to receive either a 28 day formulation (21 days of 20 mcg of ethinyl estradiol and 100 mcg of levonorgestrel followed by 7 days of placebo) or a 91 day formulation (84 days of 30 mcg of ethinyl estradiol and 150 mcg of levonorgestrel followed by 7 days of 10 mcg of ethinyl estradiol). Females using the 28 day formulation COC gained significantly less spine BMC, BMD, proximal femur BMD and total body BMC than controls not taking COCs. However, none of the differences between the 28 day COC users and controls exceeded 3%, the “non-inferiority margin” defined as clinically significant by the investigators. By contrast, there were no statistically significant differences in the percent change between the 91 day COC users and controls. At the lumbar spine, mean changes in BMD were 2.50% for controls, 2.25% for the 91 day COC group and 1.45% for the 28 day group. The study had several limitations including an attrition rate of 36%. In addition, controls were 1 year younger than COC users, introducing a potential bias since the higher rates of bone mineral accrual would be expected in younger teens. Finally, because the 91 day preparation contained higher doses of estrogen and progestin than the 28 day COC, it was not possible to determine if the sex steroid concentrations or days of active treatment accounted for differences in BMD. The authors concluded that additional research was needed to confirm their observations.

Data from these myriad studies have been combined to better understand the potential adverse effects of COCs on bone health during adolescence. A review of investigations published by 2008 concluded that COCs containing 20 mcg of ethinyl estradiol fail to provide adequate sex steroids for optimal bone accrual (24). A subsequent meta-analysis used quantitative pooling of data from individual studies to determine the effect of COCs on adolescent bone mineral accrual (25). The investigators reviewed 84 publications on the effects of COCs on BMD in teens and identified nine studies appropriate for comparison. The weighted mean difference in absolute spine BMD change at 12 and 24 months in COC users was significantly less than in controls. The magnitude of this difference was −0.02 gm/cm^2^ (95% confidence interval −0.05, 0.00), equivalent to 60% of one SD lower than rates of change in young adult controls. Gains in femoral neck BMD over 24 months also tended to be lower in COC users than controls in the four studies that examined this site. Two year changes in total hip BMD were similar in one study and significantly less in the two studies reporting these data. Gains in whole body after 12 or 24 months were also lower in COC users in two studies. The authors concluded that “evidence for potential impairment of peak spinal BMD accrual is of concern,” warranting further study in randomized controlled trials.

Several mechanisms have been proposed to explain the adverse skeletal effects of COCs on the adolescent skeleton. Low-dose COC formulations may not provide adequate estrogenic replacement (24). Alternatively, supraphysiologic doses of ethinyl estradiol suppress bone resorption necessary for bone remodeling and may inhibit periosteal apposition (25). In addition, ethinyl estradiol taken orally has been shown to inhibit IGF-1 and increase IGF binding protein 3 production, thus further limiting the availability of free IGF-1 (5). This suppressive effect is dose-related, with IGF-1 concentrations decreasing as ethinyl estradiol increases from 20 to 35 mcg (26).

Patterns of contraceptive use are often variable, with females utilizing differing COC formulations or suspending usage altogether. It remains uncertain whether reduced accrual or loss of BMD is fully reversible after COCs are stopped or result in a compromise of peak bone strength. One study found that teens (ages 14–18 at entry) taking low dose (<30 mcg EE) or standard dose (30–25 mcg EE) COC had smaller adjusted 24 month percent gains in spine and whole body BMD than non-users; mean absolute BMD change was also significantly lower for spine and whole body. Concerningly, teens who discontinued COCs continued to have smaller gains in spine BMD than non-users at 12 and 24 months after stopping (27). Additional studies in adolescents are needed to explore the extent of bone accrual after COC use.

## Transdermal Contraceptives

Data on the skeletal effects of combined estrogen-progestin transdermal contraceptives (referred to as “the patch”) are limited in teens. A pilot study compared bone density and circulating hormone levels in 5 teens (ages 16–18) using transdermal Ortho Evra^R^ (ethinyl estradiol/norelgestromin) for about 12 months with control non-users (28). There were no significant differences in IGF-1 concentrations between the groups. However, Ortho Evra^R^ users had no significant gains in whole body BMC, hip BMD, or spine BMD by DXA while controls gained 3.9, 2.7, and 2.8%, respectively. Transdermal estrogen alone (Vivelle Dot^R^) is not a form of birth control, but has been used for hormone replacement in teens with functional hypothalamic amenorrhea as discussed below (29).

## Progestin-Only Contraceptives

Progestin-only contraceptives are available as implantable capsules, intrauterine devices, oral mini pills, and intramuscular or subcutaneous injections. In general, the systemic dose of progestin delivered by all these products is relatively low except for the parenteral formulation, intramuscular depot medroxyprogesterone acetate (DMPA). As a result, there is less suppression of endogenous estrogen levels (6). In theory, the potential for adverse skeletal effects from progestin-only contraceptives other than DMPA may also be less. Regrettably, data on the short- and long-term changes in BMD with progestin-only contraceptives in adolescents is very limited with the exception of intramuscular depot medroxyprogesterone acetate (DMPA).

## DMPA

Intramuscular DMPA inhibits endogenous estrogen production resulting in lower estrogen concentrations and bone loss. This effect is dose-related, with decreases in BMD observed in adolescents (aged 12–21) given 150 mg or 104 mg every 12 weeks, but not when treated with 75 mg (30).

Bone loss has been observed in females of all ages using the standard DMPA formulation (150 mg intramuscularly every 12 weeks), with greater losses in teens than in mature women. One study comparing DMPA users aged 16–24 with those aged 25–33 found significantly greater declines in BMD at the spine BMD (−4.2 vs. −3.2%) and femoral neck (−6.0 vs. −4.2%) over a 3 year period in the younger women (31). Another prospective study compared changes in BMD in 70 DMPA users and 90 non-user controls (aged 14–18 at entry) (32). BMD decreased significantly at hip and spine but not whole body with DMPA use. The calculated annualized mean percentage change in BMD was −1.81, −0.97, and 0.73% at hip, spine and whole body, respectively, for DMPA users as compared with −0.19, +1.32, and +0.88% for non-users. Losses were greater in new users than in long-term (prevalent) users and were most rapid during the first 1–2 years. A third study also documented losses in BMD at spine, total hip or femoral neck in 98 teens (aged 12–18) studied prospectively after initiating DMPA use. BMD loss of <5% was observed in 47%, between 5 and 8% in 16% and >/= 8% in 37% (33). Magnitude of loss was related to total number of injections (duration of use).

Decreases in BMD have been shown to be largely or completely reversible once DMPA is discontinued (32, 33). Spine BMD recovered more quickly than the hip region, returning to baseline levels by 60 weeks after the last injection at the spine, 180 weeks at the femoral neck and 240 weeks at the hip (33). Recovery was more complete in those who had lost <5% BMD.

Concerns for bone loss led the Food and Drug Administration to add a black box warning to the DMPA packaging in 2004 cautioning against long-term use (>2 years). However, reproductive health providers published statements supporting the continued use of DMPA in adolescent and adult females beyond the proposed 2 year limit on DMPA use in adolescents (34) and young women (35). These experts underscored the reversible nature of bone loss once DMPA is discontinued and the lack of data to indicate a significantly increased fracture risk. They also have argued that the risk of bone loss should be weighed against the potential economic, psychosocial and skeletal effects of an unplanned teen pregnancy.

## Other Progestin-Only Contraceptives

Progestin-only mini pills are not a preferred option in an adolescent population due to a high failure rate with typical use. By contrast, long-acting reversible contraception (or LARCs) including hormonal intrauterine devices (Mirena^R^, levonorgestrel) or implants (Nexplanon^R^, etonogestrel) are prescribed increasingly in teens. Adolescent medicine experts have suggested that LARCs be considered as first-line contraceptive methods because of their low failure rate and diminished bleeding and cramping with menses (36). Data on the effects of these agents on BMD in adolescent users are very limited. A study of only 7 teens using a levonorgestrel (LNG) implant had gains in BMD over 12 months (19). Cross-sectional and longitudinal studies of adult women who had used LNG-IUDs for 7–10 years were found to have similar forearm BMD as compared with copper IUD users (37). Data from adolescent LNG-IUD users are lacking and warrant further study. However, the adverse effects on bone health are likely to be limited since there is very little systemic absorption of the progestin from the device. Consequently, there is less suppression of endogenous estrogen production. One study found mean serum concentrations of estradiol in LNG-IUD users was shown to be comparable to levels seen during the follicular phase of the normal menstrual cycle (38).

## Oral Contraceptive Use for Sex Steroid Replacement

Hormonal contraceptives have been prescribed to treat functional hypothalamic hypogonadism in females with anorexia nervosa or athletes with oligo-amenorrhea, referred to as the Female Athlete Triad (9). Skeletal health is compromised in these conditions not only by sex steroid deficiency, but by energy deficits and low IGF-1 as well. Decreased BMD accrual or loss can result, increasing the risk for stress or fragility fractures (39).

Oral contraceptives have proven insufficient to increase BMD in teens with anorexia nervosa in several studies (39–41). Similarly, there is no convincing evidence that oral estrogen/progestin therapy protects bone health in ballet dancers (42) or runners with features of the Female Athlete Triad (43, 44). However, an 18 month randomized, controlled study of 80 females with anorexia nervosa (aged 13–27 years) found that a combination of low dose COCs (20 mg ethinyl estradiol/100 mcg levonorgestrel) and oral dehydroepiandrosterone (DHEA, 50 mg/d) prevented the decrease in femoral BMD seen in the placebo group and improved measures of cross-sectional bone geometry (45).

A review of 10 studies in oligo-amenorrheic premenopausal women, most of whom had hypothalamic amenorrhea, found a positive effect on BMD in 7, no effect in 2, and a negative effect in one case report (43). These studies were limited by small cohorts (5–24 subjects per treatment group) and two included females with primary ovarian failure or unspecified causes for amenorrhea.

Findings of the largest randomized study of COCs in athletes were similarly inconclusive. The 2 year, open-label study of 100 eumenorrheic and 50 oligo/amenorrheic competitive female distance runners (aged 18–26 years) found no difference in the change in BMC and BMD or stress fractures between women randomized to a COC containing 30 mcg of ethinyl estradiol and controls not on hormonal contraceptives (44). The study was limited by attrition and by subjects choosing to start or discontinue OCPs. Only when the data were reanalyzed by actual COC use did trends show some potential benefit. Oligo-amenorrheic runners who took OC for at least 6 months gained about 1% per year in spine BMD (*P* < 0.005) and whole-body BMC (*P* < 0.005), amounts similar to those gained by runners who regained periods spontaneously and significantly greater than those in runners who remained oligo/amenorrheic (*P* < 0.05). The gains in bone with OC use were independent of changes in weight or body composition. Randomization to OC was not significantly related to stress fracture incidence, but the direction of the effect was protective in both menstrual groups (hazard ratio [95% CI] 0.57 (0.18, 1.83)], and the effect became stronger in treatment-received analyses.

Despite the lack of proven efficacy to increase BMD or reduce stress fractures, COCs are often prescribed to oligo-amenorrheic athletes (46). Guidelines from the American College of Sports Medicine and the Endocrine Society emphasize the limitations of pharmacologic therapy and underscore the importance of nutritional therapy to address bone loss in athletes with functional hypothalamic amenorrhea (9, 47, 48). The recommended treatment involves a multidisciplinary approach with medical, dietary and mental health support (48). The Endocrine Society guidelines specifically advise against the use of OCPs for the sole purpose of regaining menses or improving BMD.

The failure of COCs to improve BMD likely reflects the pathogenesis of bone fragility in anorexia nervosa and athletic amenorrhea. Replacing sex steroids alone is ineffective without addressing the energy deficit and resultant low IGF-1. In fact, estrogen administration by the non-physiologic oral route appears to further compromise already reduced IGF-1 production. COCs pass first through the liver where the exogenous estrogen suppresses IGF-1 production and stimulates production of IGF binding proteins resulting in reduced free hormone that is most bioactive. Providing estrogen by the transdermal route avoids the liver and the resulting inhibition of IGF-1 production, creating a more physiologic form of hormone replacement.

In contrast to COCs, transdermal estrogen has shown promising skeletal effects in adolescents with anorexia nervosa (49). In an 18 month study, 96 females (ages 12–18) randomized to transdermal estrogen (100 mcg of 17*B*-estradiol with cyclic oral progesterone) had significantly greater gains in spine and hip BMD Z-score than those assigned to a placebo patch and pills. None of the studies have been large enough or sufficiently long-term to examine the impact of transdermal hormone therapy on the incidence of stress or fragility fractures.

A second randomized, controlled study in 121 oligo-amenorrheic female athletes (age 14–25 years) also found skeletal benefits with transdermal estrogen (29). Changes in hip and spine BMD were measured at baseline, 6 and 12 months of therapy with a transdermal estradiol patch (100 mcg of 17*B*-estradiol changed twice weekly with oral progesterone 12 days per month), a COC (30 mcg ethinyl estradiol and desogestrel) or no hormones. Improvements in spine and femoral neck BMD Z-scores were significantly greater in those treated with the patch vs. the pill and those receiving no sex steroids. Hip BMD Z-scores increased significantly more with the patch vs. the pill groups. A subset of the participants in this study had repeated assessments of disordered eating using standardized instruments. Estrogen replacement both with the patch and the COC was associated with improvements in disordered eating measurements (50). Since addressing energy deficits is essential to improve bone health both in the Female Athlete Triad and anorexia nervosa, finding a way to alter restrictive eating is an important part of therapy. The dose of transdermal estrogen prescribed for hypothalamic amenorrhea is not sufficient to serve as contraception; sexually active teens must be reminded of the need for effective protection against unplanned pregnancy.

## Hormonal Contraceptives and Fractures

The risk of clinical bone fragility resulting from hormonal contraceptives use in adolescence remains uncertain because of a lack of adequate data on fracture incidence. Most studies have relied upon surrogate indicators of skeletal effects including BMD or bone turnover markers. These endpoints are chosen because they can be assessed in short term studies requiring smaller cohorts for adequate power. However, these surrogates are imperfect predictors of clinical bone fragility in younger individuals. There is no established “fracture threshold” based on BMD or BMC for teens or pre-menopausal females (51).

The gold standard for assessing clinical bone fragility is analysis of fracture incidence. A 2015 Cochrane review analyzed data from 14 observational studies comparing fracture rates in women with a history of premenopausal hormonal contraceptive use to non-users (52). The analysis found no association between COC use and fracture risk overall; however, subgroups of women with 10 or more prescriptions or those who used COCs for more than 10 years had an increased risk. By contrast, the authors concluded that DMPA use may increase fracture risk for current or prior users and that risk was increased with longer use. A single study found a decreased fracture risk in hormonal IUD users. There were no studies indicating an increased incidence of fractures in adolescents.

Two larger studies examining fracture rates in DMPA users have been published since that review. A population-based, case-controlled study of adult women (aged 20–44) from the United Kingdom found an increased adjusted odds ratio for incident fractures for current users (with 9–27 months exposure) or past DMPA users (with >30 months exposure) (53). The fracture risk was highest among women under age 30 with longer DMPA exposure (>/= 10 prescriptions; OR 3.04, 95% CI 1.36–6.81). Based upon these findings, the investigators cautioned against use of DMPA for more than 2 years, especially in younger women.

A subsequent retrospective study from a large health care group in the United States compared non-traumatic fracture rates in 308,876 women (ages 12–45) who began use of COC, progestin-only pills, DMPA or IUDs (copper or levonogesterol) between 2005 and 2015 (54). Women with more than 2 years of cumulative use of COCs or progestin-only contraceptive pills had lower fracture risk compared to those who had not used OCPs [adjusted HR 0.85 (95% CI 0.76–0.960)] or used other methods [0.88 (95% CI 0.80–0.97)]. By contrast, the fracture rate was greater among recent (within 2 years) DMPA users and those with more than 2 years of cumulative use when compared to women who had never used DMPA [adjusted HR 1.15 (95% CI 1.01–1.3)]. Fracture rates were higher among adolescents and women age 50 or older (9.0 and 8.1/1,000 person-years, respectively). Fracture risk was not increased in women whose last DMPA injection was more than 2 years earlier. The investigators concluded that since the fracture risk with DMPA was low (two fractures per 1,000 person-years), there should not be an absolute contraindication to continuing the injections beyond 2 years.

## Knowledge Gaps

More research is needed to address the limitations of studies to date on the skeletal effects of hormonal contraception in teens. Adolescent cohorts have been small, COC formulations have varied and duration of follow up for COC and DMPA users has been too short to assess fracture incidence. Not all studies controlled for key confounding variables such as gynecological age, smoking, physical activity, race-ethnicity, body mass index, or nutrition. These are important consideration since teens who are sexually active earlier may differ from their abstinent peers in terms of lifestyle and maturity.

Efforts at meta-analysis are laudable (25) but still leave unanswered several questions. How reversible are the changes in bone accrual once the medication is discontinued? Recovery of bone loss after DMPA has been observed but is there full recovery of bone mineral accrual after COCs are discontinued? Is the evidence sufficient to establish 30 mcg of ethinyl estradiol as the optimal dose to protect bone health in teens? Is the 90 day COC dosing more beneficial to bone than the 28 day cycling? What are the skeletal effects of LARCs with short- and long- term use? Does hormonal contraceptive use increase the lifetime risk of fragility fractures. Will the reassuring long term data from mature women on fracture rates with COC use prove to be similar for those who begin use of hormonal contraceptives as teens?

Additional studies in girls with normal reproductive function are clearly needed to address these questions. Designing this research involves myriad challenges including selection of appropriate controls. Randomization to a placebo arm would be unethical given the goal of pregnancy prevention. Controlling for differences in clinical characteristics is key since teens who engage in sexual activity at a younger age are likely to differ from abstinent peers in alcohol or recreational drug use, smoking, physical activity, and other lifestyle variables. Despite these challenges, this research is needed to counsel teens optimally about the impact of hormonal contraception on their lifetime bone health.

Further studies are warranted as well to determine the skeletal benefits of very low dose or transdermal estrogen therapy for hypothalamic amenorrhea. The optimal sex steroid replacement to protect bone health in primary gonadal failure remains to be determined as well. Investigations with HRpQCT will enhance insights into how sex steroids influence the microarchitecture contributing to bone strength.

Until these questions are addressed with future studies, counseling teens about hormonal contraceptives must be based upon current knowledge (36). For teens seeking effective birth control, the potential skeletal risks of any method must be weighed against the benefits of avoiding the health, financial and social costs of an unplanned pregnancy. LARCs have been endorsed as a first line method of contraception because of its low failure rate and less menstrual bleeding (36). For teens opting to use COCs, preparations with 30 mcg of ethinyl estradiol are recommended over lower dose preparations. Any teen choosing COCs or DMPA should be counseled on healthy lifestyle habits, including smoking cessation, activity, and adequate calcium and vitamin D intake (55). Hormonal contraceptives are increasingly used for indications other than contraception, such as acne, hirsutism, and dysmenorrhea. Weighing the risks and benefits for these teens may be more challenging.

## Conclusions

The skeletal effects of some forms of hormonal contraception are greater in adolescent females than in mature women. In particular, COCs and DMPA use has been associated with smaller gains or loss of bone mass during this critical period for acquiring bone strength. Data on the skeletal effects of progestin-only contraception including LARCs are very limited. Whether the reported changes in bone mineral density or bone turnover markers predict peak bone strength and lifetime fracture risk remains uncertain. More research is needed to address the long-term effects of all forms of hormonal contraception in the adolescent to allow for accurate and informative counseling.

## Author Contributions

LB was responsible for the literature review and writing of this review.

## Conflict of Interest

The author declares that the research was conducted in the absence of any commercial or financial relationships that could be construed as a potential conflict of interest.
